# Mediastinitis and sternal prosthesis infection successfully treated by minimally invasive omental flap transposition

**DOI:** 10.1186/1749-8090-8-30

**Published:** 2013-02-25

**Authors:** Valentina Tassi, Silvia Ceccarelli, Jacopo Vannucci, Francesco Puma

**Affiliations:** 1 Thoracic Surgery Unit, University of Perugia Medical School, Ospedale S. Maria della Misericordia, 06134, Perugia, Italy

**Keywords:** Mediastinum, Chest wall, Omental flap, Surgery, Complications

## Abstract

Purulent mediastinitis is a possible serious complication after mediastinal surgery. We report the case of a localized sternal plasmocytoma treated by sternectomy and prosthetic repair, who needed a second surgery for a fistulizing mediastinitis. Five months earlier, in another Hospital, the patient underwent sternal resection and reconstruction with a “sandwich” prosthesis (Methyl-methacrylate and Marlex mesh). Suppurative mediastinitis occurred and septic shock resolution was observed after the spontaneous opening of a mediastinal cutaneous fistula. After referring to our Unit the patient underwent extensive local and systemic preparation and nutritional support; the infected prosthesis was then removed and the gap filled by a laparoscopically-prepared omental flap. Adequate preoperative management, removal of any infected material and minimally invasive omental flap transposition allowed the successful treatment of this life-threatening condition.

## Background

Purulent mediastinitis secondary to chronic prosthetic infection after sternectomy is a very difficult problem to resolve. The goals of surgical treatment are fast removal of the infected prosthetic material, mediastinal debridement and mediastinal filling with well vascularized tissues. Surgical trauma should be as mild as possible for the usually weak patient’s general conditions [1,2].

## Case presentation

We describe the case of a 73-year-old caucasian male with purulent mediastinitis and prosthetic infection after subtotal sternectomy.

Five months earlier, the patient underwent surgery for solitary plasmocytoma in another hospital; chest wall reconstruction was achieved by a Marlex methyl-methacrylate sandwich prosthesis. Two days after surgery, the wound was re-explored to control bleeding from the operative site.

At the admittance to our hospital the patient was in a bad clinical condition, with intermittent fever, dyspnea and anterior chest pain. His comorbidities included chronic C virus hepatitis, monoclonal gammopathy and multinodular goiter. Blood sampling revealed an increase in white blood cells, erythrocyte sedimentation rate (ESR) and C-reactive Protein (CRP). Local examination showed an erythematous, swollen, indurated sternal wound with a chronic, purulent fistula opening at the lower third of the surgical incision.

**Figure 1:**
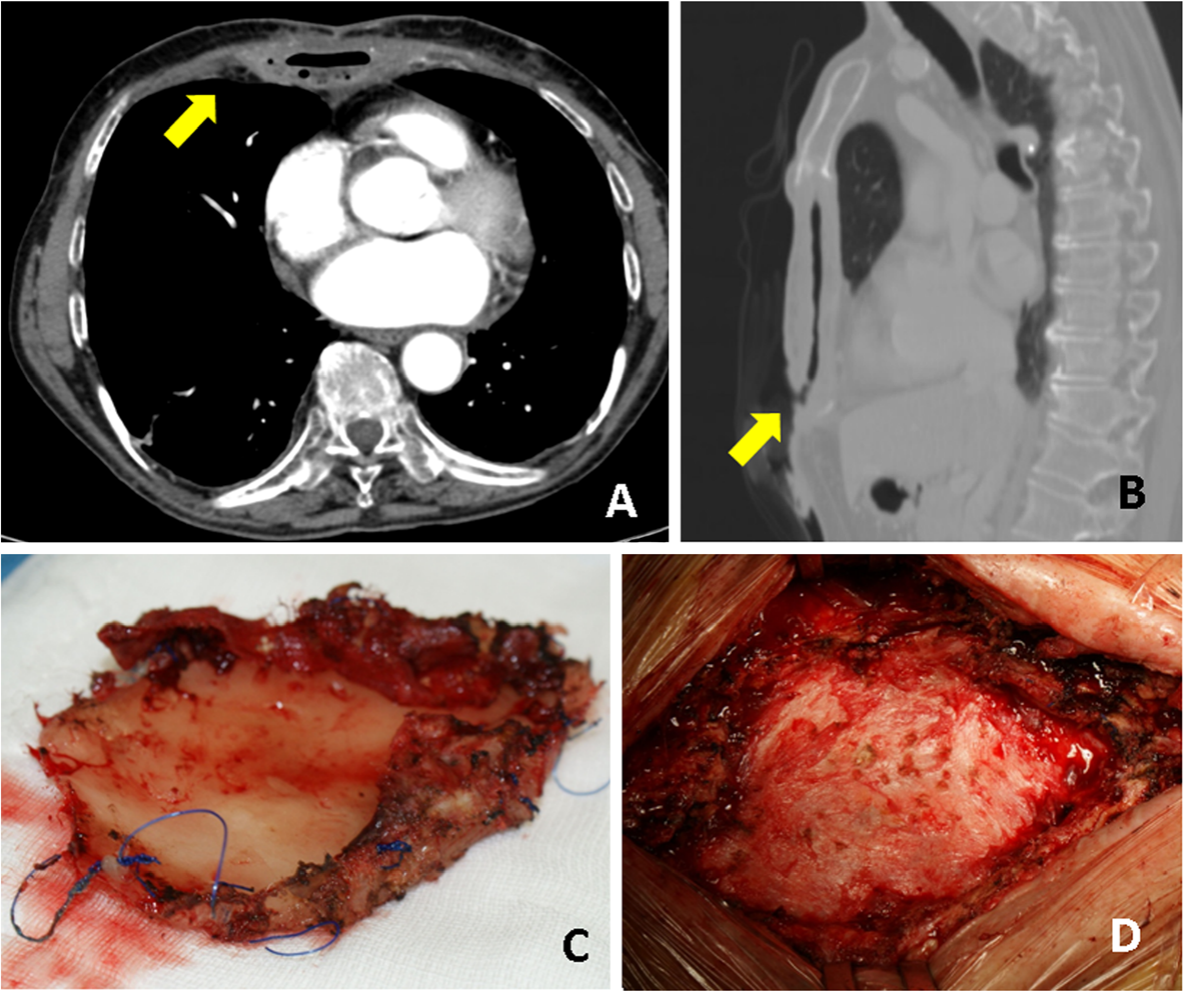
A-B) Computed tomography scan shows the infected prosthesis surrounded by multiple leveled hyperdense fluid (arrows) C) Methyl-methacrylate plate after removal; D) Intraoperative finding after prosthesis removal.

A computed tomography (CT) scan showed semi-solid inflammatory tissue with multiple air levels involving the mediastinal surface of the sternal prosthesis and the pericardium (Figure 1A-B). Microbiological culture of wound secretions revealed a Multidrug Resistant Pseudomonas Aeruginosa infection. After 40 days of specifically targeted antibiotic intravenous therapy, daily dressing and mediastinal irrigation with antibacterial solution, no wound healing was obtained nor culture negativization. Nutritional status improvement was achieved through a hypercaloric, hyperproteic diet.

**Figure 2:**
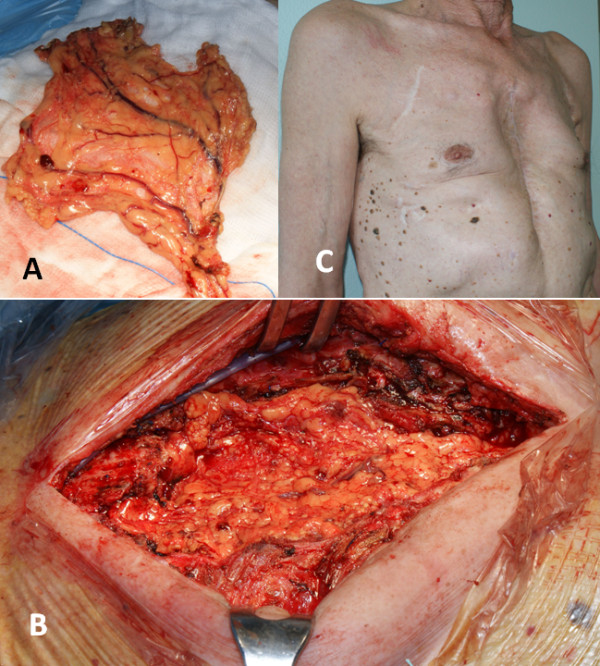
A) Trans-diaphragmatic omental flap transposition, B) Final wound appearance C) External examination 6 months after surgery.

Under general anesthesia with a single lumen oro-tracheal intubation, we laparoscopically prepared an omental flap, transposed at the thoracic level through a minimal diaphragmatic anterior incision (Figure 2A). The technique of laparoscopic omental flap preparation has been described in detail elsewhere [3]. A median sternotomy incision on the existing scar was then carried out, encircling the edges of the fistula. The sandwich prosthesis was clearly infected although the deep layer of the Marlex mesh was partially incorporated into the mediastinal tissues. We entirely removed the superficial mesh and the methyl-methacrylate plate while the deep incorporated Marlex mesh was partially left in place (Figure 1C). An accurate mediastinal debridement (Figure 1D) was performed with Volkmann’s spoon. The omental flap was finally gently placed to fill the defect, fixed with interrupted sutures to the wound edges (Figure 2B). Two Jackson-Pratt drains were positioned beneath the pectoralis major muscles, which were partially detached from the chest wall and advanced medially with a tension-free suture.

Patient was discharged on postoperative day 10 and received intravenous antibiotic therapy for another 2 weeks. Drains were removed on postoperative day 4; ESR and CRP decreased towards baseline values within 47 days. The wound healed well without significant flail chest (Figure 2C). A CT scan, performed 3 months after surgery, did not demonstrate radiographic signs of infection nor residual substernal airspace.

## Discussion

A rigid plaque as methyl-methacrylate in combination with two layers of Marlex mesh has been largely employed to repair large defects after sternectomy. The possible infection of this type of prosthesis is already known [4] and has led us and others to investigate alternative techniques [4-6]. Infection of a prosthetic reconstruction after sternectomy is challenging to resolve and requires prompt removal of the prosthetic material. The use of vascularized tissue is needed for infection control, while flail chest is generally a minor problem for the mediastinal stiffness secondary to post inflammatory fibrosis. An omental flap is theoretically preferable to muscle flaps in these circumstances due to its superior resistance to infection and because it allows a better filling of the deepest mediastinal recesses, thus avoiding the persistence of an empty space [1-3]. On the other hand, muscle flaps are more commonly used for the lower surgical trauma. The greatest disadvantages of the omentum for treating postoperative sternal osteomyelitis are linked to the laparotomy which is responsible for postoperative ileus (delayed oral nutrition) and postoperative pain (impairment of ventilatory dynamics, mucus retention, possible respiratory infections). The possibility of harvesting the omental flap by a laparoscopy is a technically easy procedure, that allows the use of such procedure even in severely compromised patients.

Therapeutic alternatives not entailing the removal of the infected prosthesis are to be considered unreliable. In fact sepsis resolution can be achieved only after the thorough removal of all the infected autologous and prosthetic tissues [7].

## Conclusion

•Removal of an infected sternal prosthesis is the key for the successful treatment.

•Intrathoracic omental flap transposition is preferable to muscle flap but entails an heavier surgical trauma.

•Laparoscopically-prepared omental allows to take advantage from the favorable properties of the omentum, without further significant surgical trauma.

## Consent

“Written informed consent was obtained from the patient for publication of this Case report and any accompanying images. A copy of the written consent is available for review by the Editor-in-Chief of this journal.”

## Abbreviations

ESR: Erythrocyte sedimentation rate;CRP: C-reactive protein;CT: Computed tomography

## Competing interest

All authors declare that they have no competing interest.

## Authors’ contribution

VT, SC and FP wrote the article, JV and SC collected the clinical information, JV selected the images; VT and SC analyzed the English Literature. FP drafted the final manuscript. All authors approved the final manuscript to be published.
